# Expression and Localization of Paneth Cells and Their α-Defensins in the Small Intestine of Adult Mouse

**DOI:** 10.3389/fimmu.2020.570296

**Published:** 2020-10-13

**Authors:** Kiminori Nakamura, Yuki Yokoi, Rie Fukaya, Shuya Ohira, Ryuga Shinozaki, Takuto Nishida, Mani Kikuchi, Tokiyoshi Ayabe

**Affiliations:** ^1^ Innate Immunity Laboratory, Department of Cell Biological Science, Graduate School of Life Science, Hokkaido University, Sapporo, Japan; ^2^ Innate Immunity Laboratory, Department of Cell Biological Science, Faculty of Advanced Life Science, Hokkaido University, Sapporo, Japan

**Keywords:** Paneth cell, alpha-defensin, cryptdin, Institute of Cancer Research mouse, innate immunity, germ-free mouse

## Abstract

Paneth cells contribute to intestinal innate immunity by sensing bacteria and secreting α-defensin. In Institute of Cancer Research (ICR) mice, α-defensin termed cryptdin (Crp) in Paneth cells consists of six major isoforms, Crp1 to 6. Despite accumulating evidences that α-defensin functions in controlling the intestinal microbiota, topographical localization of Paneth cells in the small intestine in relation to functions of α-defensin remains to be determined. In this study, we examined the expression level of messenger RNA (mRNA) encoding six Crp-isoforms and Crp immunoreactivities using singly isolated crypts together with bactericidal activities of Paneth cell secretions from isolated crypts of duodenum, jejunum, and ileum. Here we showed that levels of Crp mRNAs in the single crypt ranged from 5 x 10^3^ to 1 x 10^6^ copies per 5 ng RNA. For each Crp isoform, the expression level in ileum was 4 to 50 times higher than that in duodenum and jejunum. Furthermore, immunohistochemical analysis of isolated crypts revealed that the average number of Paneth cell per crypt in the small intestine increased from proximal to distal, three to seven-fold, respectively. Both Crp1 and 4 expressed greater in ileal Paneth cells than those in duodenum or jejunum. Bactericidal activities in secretions of ileal Paneth cell exposed to bacteria were significantly higher than those of duodenum or jejunum. In germ-free mice, Crp expression in each site of the small intestine was attenuated and bactericidal activities released by ileal Paneth cells were decreased compared to those in conventional mice. Taken together, Paneth cells and their α-defensin in adult mouse appeared to be regulated topographically in innate immunity to control intestinal integrity.

## Introduction

A monolayer of intestinal epithelial cells is the largest surface exposed to various microbes. Mucosal immunity on the intestinal surface plays a pivotal role in host defense. Paneth cells, one of epithelial cell lineages in the small intestine, reside at the base of crypts of Lieberkühn and have apically oriented secretory granules which contain high levels of antimicrobial peptides, α-defensins (1, 2). Paneth cells secrete granules containing α-defensins when exposed to bacteria, bacterial antigens, cholinergic stimuli or certain nutrients, and the secreted α-defensins elicit potent microbicidal activities against pathogens (3–5). Paneth cell α-defensins are actively involved in the innate enteric immunity and maintain intestinal homeostasis by controlling the intestinal microbiota to prevent dysbiosis (6–10). In addition, Paneth cells provide survival signals to crypt intestinal stem cells, crypt-base columnar stem cells, and create stem cell niche responsible for regenerating entire lining of small intestinal epithelial cells (11).

α-Defensin in mouse, termed cryptdin (Crp), is a major microbicidal constituent of mouse Paneth cell granules (12, 13). Paneth cells contribute the innate enteric immunity by sensing bacteria and releasing microbicidal activities mostly by activated Crps at effective concentrations (3). Paneth cells contain six Crp isoforms in Institute of Cancer Research (ICR) mouse, and Crp1, 2, 3, and 6 are considered as Crp1-like family with high homogeny of the primary structure (14–17). Among Crps, Crp4 is known to elicit the most potent bactericidal activity *in vitro*, and different functions of six Crp isoforms have been discussed in previous reports. Crp4 is the most bactericidal against *Escherichia coli* as well as *Staphylococcus aureus* (12). In contrast, Crp2 and Crp3 have potent killing activities against *Giardia lamblia* trophozoites, whereas Crp1 and Crp6 have less effect (18). It has been known that Crps show site-specific distribution in the messenger RNA (mRNA) expression in the small intestine. Crp4 mRNA expression is known to be restricted mostly in the ileum (14). A human Paneth cell α-defensin, HD5 is known to have topographic differences in their gene expressions in the small intestinal tissue (19, 20). However, precise special distributions of Paneth cells and their α-defensins in entire mouse small intestine remain to be determined. Furthermore, bactericidal activities released by Paneth cells in different anatomical sites in the small intestine have not been reported and Paneth cell α-defensin expression and function in germ-free mouse remain controversial.

In this study, we analyzed the expression and localization of α-defensins in the adult mouse small intestine by analyzing mRNA expression of six Crp isoforms, Crp immunohistochemistry, and bactericidal activities of Paneth cell secretions using isolated crypts from different anatomical sites of the small intestine. We showed that Paneth cells in the small intestine are specially regulated from duodenum to ileum along with their Crps and revealed that released bactericidal activities by Paneth cells are also regulated in the small intestine consistent with the number of Paneth cells. Furthermore, we revealed that in germ-free mice, bactericidal activities released by ileal Paneth cells are reduced due to decrease of Crp expression. This study reveals anatomical, histological features of mouse Paneth cells and α-defensins, and gives additional insights into the innate enteric immunity.

## Methods

### Mice

Cr1j:CD-1 ICR (ICR) adult male conventional and germ-free mice were purchased from Charles River Laboratories Japan, Inc. and propagated at Hokkaido University. All mice were housed under conventional condition maintained under a 12 h light/dark cycle with water and food provided *ad libitum*. All animal experiments in this study were conducted after obtaining approval from the committee on Animal Care and Use at Hokkaido University in accordance with Hokkaido University Regulations of Animal Experimentation.

### Preparation of Mouse Isolated Crypts and Paneth Cells

Intact crypts were isolated from mouse small intestine by our previously reported method (3). Small intestine was resected from adult ICR conventional and germ-free mice, and duodenum, jejunum, and ileum were obtained, soaked them with 30 mM EDTA in Ca^2+^/Mg^2+^-free phosphate-buffer saline (PBS-) with vigorous vibration for every 5 min to separate six fractions. After centrifuge, each fraction was replaced with fresh ice-cold PBS-. Individual crypts were transferred to siliconized microfuge tubes using capillary pipettes. In addition, we collected a crypt-rich pool with more than 80% crypt purity by estimating crypt numbers using hemocytometer. Single crypt from each site of the small intestine was isolated by using glass pipette into the microtube under phase-contrast microscopy (x400) and collected at −80°C (21).

### Extraction and Reverse Transcription Reaction of Single-Crypt RNA

Total RNA 100 ng of singly isolated duodenal, jejunal, and ileal crypts were obtained (22). Reverse transcription reaction was conducted on the total RNA for 30 min at 55°C and 5 min 85°C (Transcriptor First Strand cDNA Synthesis Kit, Roche) using anchored-oligo (dT)_18_ primer and transcriptor reverse transcriptase, and synthesized single strand complementary DNA (cDNA).

### Real-Time PCR

Using cDNA 5 ng obtained from an isolated crypt from duodenum, jejunum, or ileum as templates, real-time PCR was conducted (LightCycler480 SYBR Green I Master Kit, Roche) using each Crp (Crp1-Crp6)-specific primer and glyceraldehyde-3-phosphate-dehydrogenase (GAPDH)-specific primer (**Supplementary Table 1**) and SYBR Green I probe (Roche, LightCycler480) (n = 10). The PCR was performed three steps of 10 s at 95°C denature, 10 s at 63°C annealing, 12 s at 73°C extension for 50 cycles after a 5 min-pre-heat at 95°C. The amplification curve of the PCR product was obtained (23), and confirmed that it was an objective product from the size that compared the marker by the 2% agarose gel electrophoresis including the ethidium bromide. Amplified PCR products were further determined by direct DNA sequencing. Furthermore, we confirmed that there was no non-specific amplification by the fusion curve analysis. Agarose gel (2%) electrophoresis was conducted using the PCR products and GAPDH to obtain standard curve, cut gels of target bands, and purified each cDNA (QIAEXIIGel Extraction Kit) (n = 4 for each) with dilution system of 10^3^ to 10^6^ copies of the cDNA, and enforced real-time PCR on similar PCR condition. The copy number of Crp1~Crp6 and GAPDH were calculated. The formula shown below was used to calculate the cDNA amount 　(primer bp) × 10^−9^/9.12 μg = 10^5^ cDNA copy. A calibration curve was obtained from the average of the obtained CP values, and the copy numbers of Crp1 to Crp6 and GAPDH mRNA were calculated and absolute quantification was performed (n=10 for each). The efficiency showing PCR efficiency was 1.6 or more.

### Histological and Immunohistochemical Analysis

For hematoxylin-eosin (HE) staining, the 4% paraformaldehyde-fixed duodenum, jejunum, and ileum from conventional and germ-free mice were embedded in paraffin and cut into 4 μm-thick sections. Then, sections were placed on glass slides and stained by hematoxylin and eosin. In addition, 4% paraformaldehyde-fixed paraffin-embedded sections were cut in serial sections and were immunostained using the following primary antibodies: mouse anti-Crp1 (77-R63) and mouse anti-Crp4 (74-4). The crypt-rich pool and the fraction of intestinal epithelium cells from duodenum, jejunum and ileum from conventional mice were fixed with 2% paraformaldehyde for 40 min at room temperature, and blocked with 0.01% normal horse serum for 30 min. Then, polyclonal anti-Crp1 antibody (rabbit-IgG) which react Crps1, 2, 3, and 6, polyclonal anti-Crp4 antibody (rabbit-IgG) which only react Crp4, and polyclonal anti-lysozyme antibody (rabbit, Dako) were reacted for 60 min. Alexa 488 Fluor (goat anti-rabbit IgG H+L, Invitrogen) as secondary antibody for Crps antibody and rhodamine phalloidin (F-actin, Invitrogen) were reacted for 60 min at 4°C. After nucleus staining by 4’,6-diamidino-2-phenylindole (DAPI) for 5 min, adhere to the cover glass which coated Cell-Tak (Corning) for 20 min, and embedded to the slide glass using Fluoromount (Diagnostic BioSystems). Using confocal microscopy (LSM510, Carl Zeiss and A1, Nikon), the samples of duodenal, jejunal, and ileal crypts and isolated Paneth cells were analyzed (n =10 for each).

### Collection of Paneth Cell Secretion and Bactericidal Assay

Individual isolated crypts from conventional or germ-free mice were incubated in either 30 μl of PBS- or PBS-containing 1,000 bacterial colony-forming unit (CFU)s of *Salmonella typhimurium* per crypt for 30 min at 37°C (n = 3 each). Cellular components were deposited briefly by centrifugation, and supernatants were transferred to sterile microfuge tubes and stored at –20°C as control supernatants and secretions with bacterial exposure. Then, 5 μl of the collected samples were incubated with 1 x 10^3^ CFU of *S. typhimurium* (3, 24) for 1 h at 37°C. Surviving bacteria were determined by plating on nutrient agar and counting colony numbers after growth for overnight at 37°C. Bacterial cell killing as the percentage relative to bacteria incubated PBS- alone were determined.

### Statistical Analysis

Data were shown in mean ± standard deviation (SD). One-way ANOVA and Tukey *post-hoc* tests were used for statistical analyses and considered p < 0.05 as statistically significant.

## Results

### Quantification of Cryptdin Gene Expression in the Isolated Crypt From Duodenum, Jejunum, and Ileum of Adult Conventional Mice

**Figure 1:**
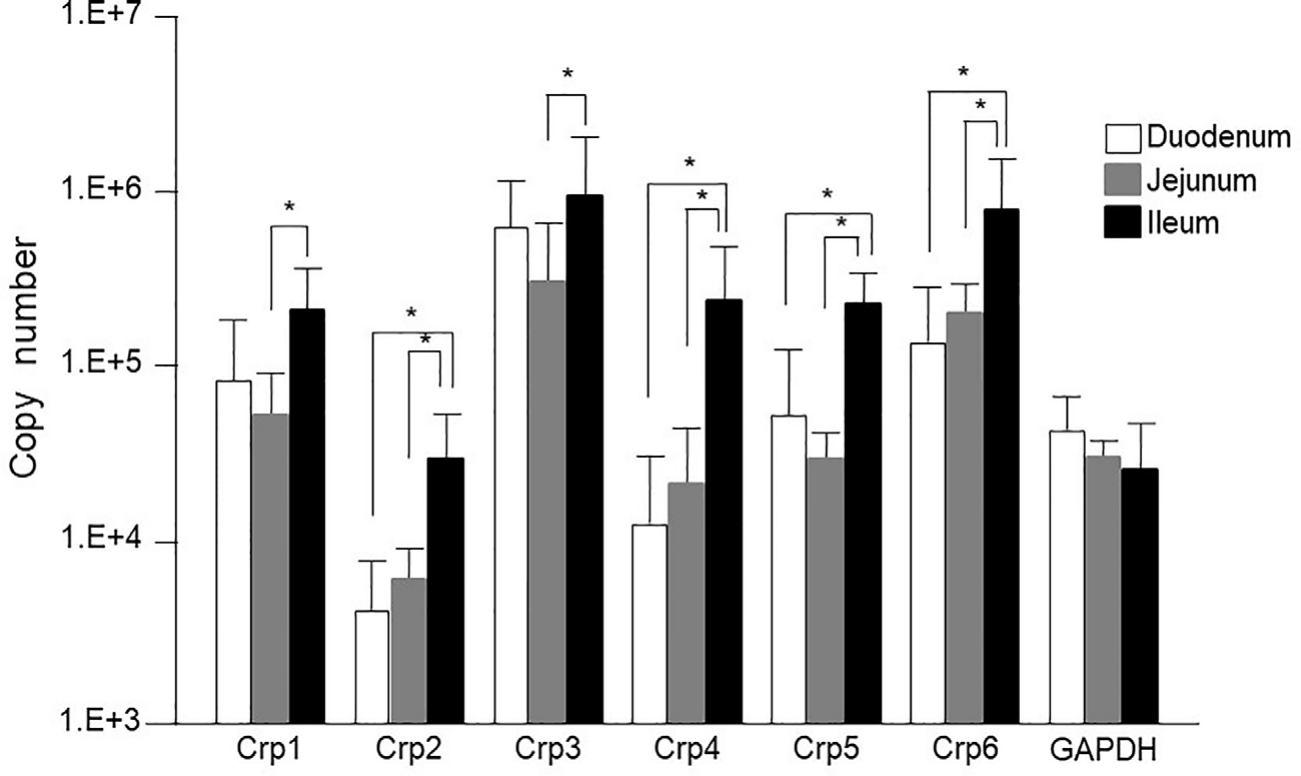
Cryptdin gene expression levels in the isolated single crypt of duodenum, jejunum, and ileum. Crp isoform messenger RNA (mRNA) copy numbers in duodenum, jejunum, and ileum at single-crypt level (n = 10 for each, mean ± SD, *p < 0.05).

First, we measured Crp isoform mRNA expression at single-crypt level by using single-crypt derived total RNA of conventional mice. The mRNA expressions of each Crp isoform in the individual single-crypt RNA from duodenum, jejunum, and ileum were Crp1; 8 x 10^4^, 5 x 10^4^, and 2 x 10^5^, Crp2; 3 x 10^3^, 5 x 10^3^, and 3 x 10^4^, Crp3; 5 x 10^5^, 3 x 10^5^, and 1 x 10^6^, Crp4; 1 x 10^4^, 2 x 10^4^, and 2 x 10^5^, Crp5; 6 x 10^4^, 3 x 10^4^, and 2 x 10^5^, and Crp6; 1 x 10^5^, 2 x 10^5^, and 8 x 10^5^, respectively. There was significantly higher gene expression for each Crp isoform in the ileum compared to that in the jejunum (**Figure 1**). In addition, there were significantly higher gene expression for Crp2, Crp4, Crp5, and Crp6 in the ileum compared to those in the duodenum. In contrast, no significant difference was shown on each Crp isoform gene expression between duodenum and jejunum. The gene expression of Crp3 was highest and Crp2 was lowest among Crp isoforms from duodenum to ileum. GAPDH gene expressions in duodenum, jejunum, and ileum were 4 x 10^4^, 3 x10^4^, and 3 x 10^4^, respectively, and no significant differences were observed.

The mRNA expression of each Crp isoform in single-crypt obtained from duodenum, jejunum, and ileum was calculated as the ratio *versus* gene expressions of GAPDH in single-crypt from each corresponding site. The Crp/GAPDH ratios for duodenum, jejunum, and ileum were Crp1; 3.4, 2.8, and 14.2, Crp2; 0.1, 0.2, and 2.3, Crp3; 24.4, and 13.9, 111.6, Crp4; 0.5, 1.5, and 23.7, Crp5; 2.7, 1.3, and 18.6, and Crp6; 14.2, 11.5, and 74.1, respectively. All Crp isoform mRNA expression ratios were remarkably higher in ileum than duodenum and jejunum (**Supplementary Figure 1A**).

The gene expression of Crp1-6 in the single-crypt of the ileum was greater 4.2, 19.7, 4.6, 48.3, 6.9, and 5.2 times compared to those in the duodenum, respectively. The gene expression of Crps in the ileum was increased four times of Crp-1 to 48 times of Crp-4 than those in the duodenum. The mRNA expressions of Crp1 and Crp4 were 5 and 16 times higher in the ileum than those in the jejunum, respectively. The difference of each Crp isoform gene expression was the smallest in Crp1 and the greatest in Crp4 (**Supplementary Figure 1B**).

### Immunolocalization of Cryptdin and Number of Paneth Cells in the Isolated Crypts of Adult Conventional Mice

**Figure 2:**
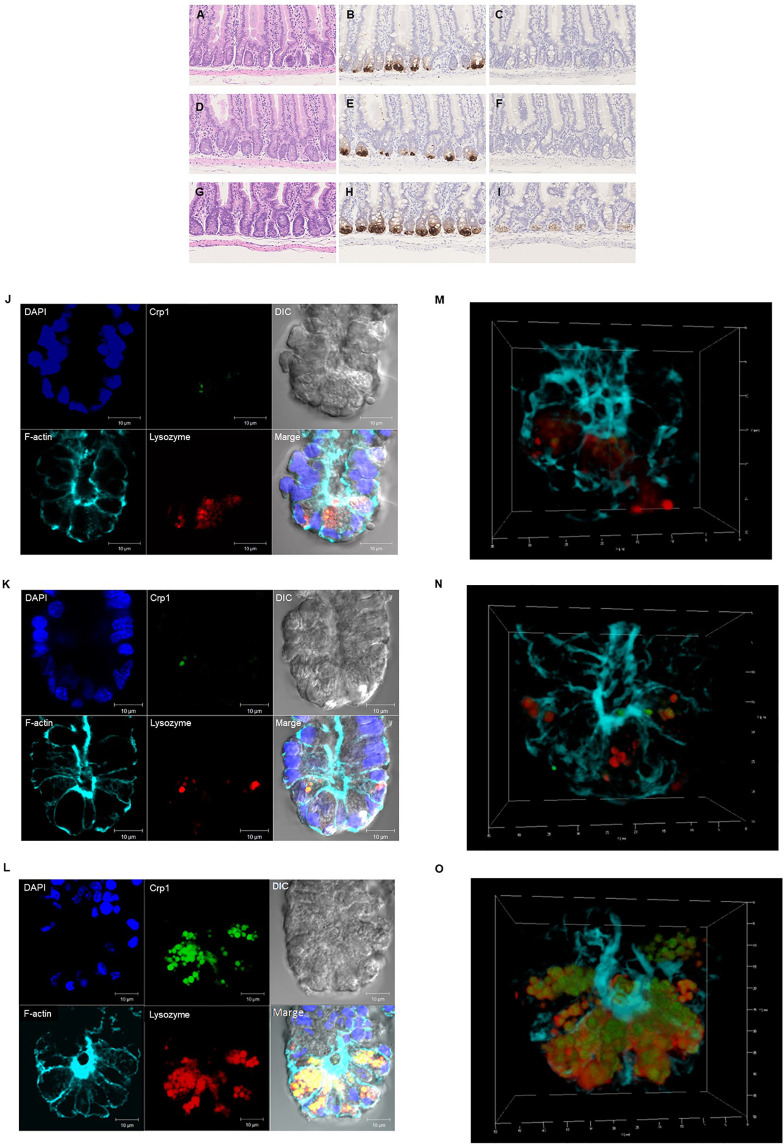
Histological and immunohistochemical analyses of the small intestine. Hematoxylin-eosin staining of the duodenum **(A)**, jejunum **(D)**, and ileum **(G)**. and immunohistochemical analysis of Crp1 in the duodenum **(B)**, the jejunum **(E)**, and the ileum **(H)** and Crp4 in the duodenum **(C)**, the jejunum **(F)**, and the ileum **(I)** of mouse small intestine. Immunohistochemical analyses of isolated single crypt from duodenum **(J)**, jejunum **(K)**, and ileum **(L)** using confocal microscopy. Representative images of each site with Crp1 and lysozyme staining together with 4’,6-diamidino-2-phenylindole (DAPI), F-actin, and differential interference contrast (DIC) were shown. The same crypt observed in **(J–L)** with Crp1, lysozyme, and F-actin were shown by 3D images in **(M–O)**, respectively. A representative image of 10 crypts was shown.

**Figure 3:**
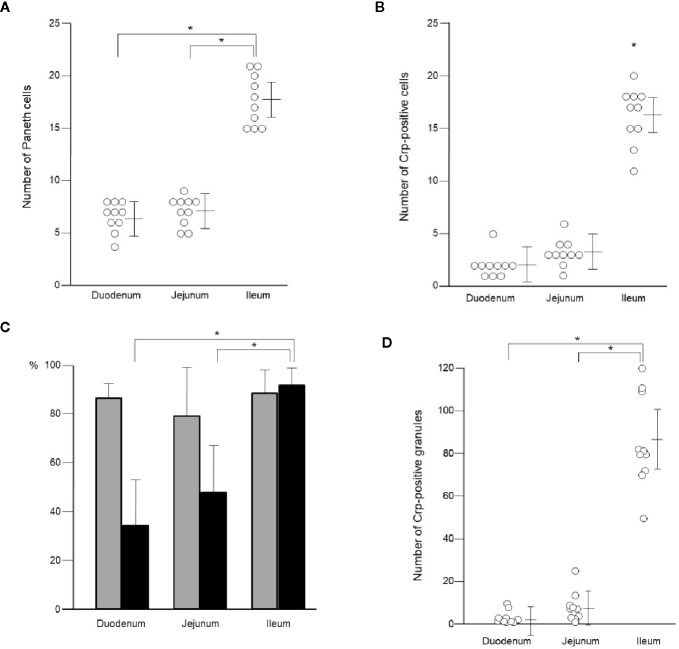
Number of Paneth cells and their granules in single crypt of duodenum, jejunum, and ileum. Number of Paneth cells in single crypt of duodenum, jejunum, and ileum **(A)**. Number of Crp-positive cells in single crypt of duodenum, jejunum, and ileum **(B)**. Percentage of lysozyme-positive (gray column) and Crp-positive (black column) Paneth cells in single crypt of duodenum, jejunum, and ileum **(C)**. Number of Crp-positive granules in single-crypt of duodenum, jejunum, and ileum **(D)**. n = 10 for each, mean ± SD, *p < 0.05.

Paneth cells could be recognized with HE staining of the small intestine of conventional mice, showing eosin-positive granules in the cytoplasm (**Figures 2A–I**). Crypts of duodenum, jejunum, and ileum were identified by the microscope with Nomarski method (DIC image) showing Paneth cells which reside at the crypt base with dense intracellular granules. The size of the crypt was almost same among duodenum, jejunum, and ileum. The Paneth cell granules were spheres of the 0.5 ~2 μm, and the number of granules in one Paneth cell was from 3 to 30. Crp1 and lysozyme were immunostained with granules of the Paneth cells (**Figures 2J–L**). Three-dimensional structure of Paneth cells in the isolated single crypts by the image of phalloidin indicating cytoskeleton and DAPI indicating nucleus were shown in **Figures 2M–O**. The number of Paneth cells in a single crypt from duodenum, jejunum, and ileum was 6.6 ± 1.3, 7.1 ± 1.4, and 17.7 ± 2.5, respectively. Paneth cells were significantly rich in the ileum than those in duodenum and jejunum (**Figure 3A**). No significant difference in the Paneth cell number was observed between duodenum and jejunum.

Crp1 antibody reaction was restricted to the granule in the Paneth cell. However, some Paneth cells were negative for Crp. The numbers of Paneth cell (Crp-positive Paneth cell) in the duodenum, the jejunum, and the ileum were 2.0 ± 1.2, 3.2 ± 1.3, and 16.2 ± 2.7, respectively per one crypt, indicating that there are significantly abundant Crp-positive Paneth cells in ileum compare to duodenum or jejunum (**Figure 3B**). In contrast, no significant difference was shown in numbers of Paneth cell between duodenum and jejunum. The Crp-positive ratios in Paneth cells in duodenum, jejunum, and ileum were 33.9, 45.5, and 91.5%, respectively, indicating that ileal Paneth cell contains significantly greater Crps than duodenum and jejunum (**Figure 3C**).

Because α-defensin is packed in Paneth cells granules, we further counted Crp-poisitive granule numbers in Paneth cells. Numbers of Crp-positive granules in single crypt in duodenum, jejunum, and ileum were 3.4 ± 3.1, 7.9 ± 6.2, and 85.1 ± 21.5, respectively. There were significantly abundant numbers of Crp-positive granules in ileum compere to duodenum and jejunum (**Figure 3D**). Lysozyme, an antimicrobial protein, was also known to locate only in Paneth cell granules in intestinal epithelial cells. Therefore, we further determined numbers of Paneth cells containing lysozyme-positive granules in duodenum, jejunum, and ileum, and the numbers were 5.6 ± 1.6, 5.6 ± 1.6, and 15.6 ± 3.0, respectively, indicating significantly abundant lysozyme-positive Paneth cells in ileum. The lysozyme-positive ratios of Paneth cells were similar in entire small intestine; 86.6% in duodenum, 79.8% in jejunum, and 88.1% in ileum (**Figure 3C**). We further showed that the number of lysozyme-positive granules in Paneth cells in single-crypt from duodenum, jejunum, and ileum were 21.0 ± 9.2, 24.0 ± 11.9, and 82.0 ± 26.7, respectively. The numbers of granules which showed Crp1/lysozyme-double positive in duodenum, jejunum, and ileum were 2.1 ± 3.0, 5.6 ± 7.4, and 68.5 ± 21.9, respectively. Taken together, Paneth cell numbers in the small intestinal crypts of conventional mice are greatly increased from proximal toward distal small intestine.

### Immunolocalization of Cryptdin and Number of Paneth Cells in Adult Germ-Free Mice and Bactericidal Activities of Paneth Cell Secretions

**Figure 4:**
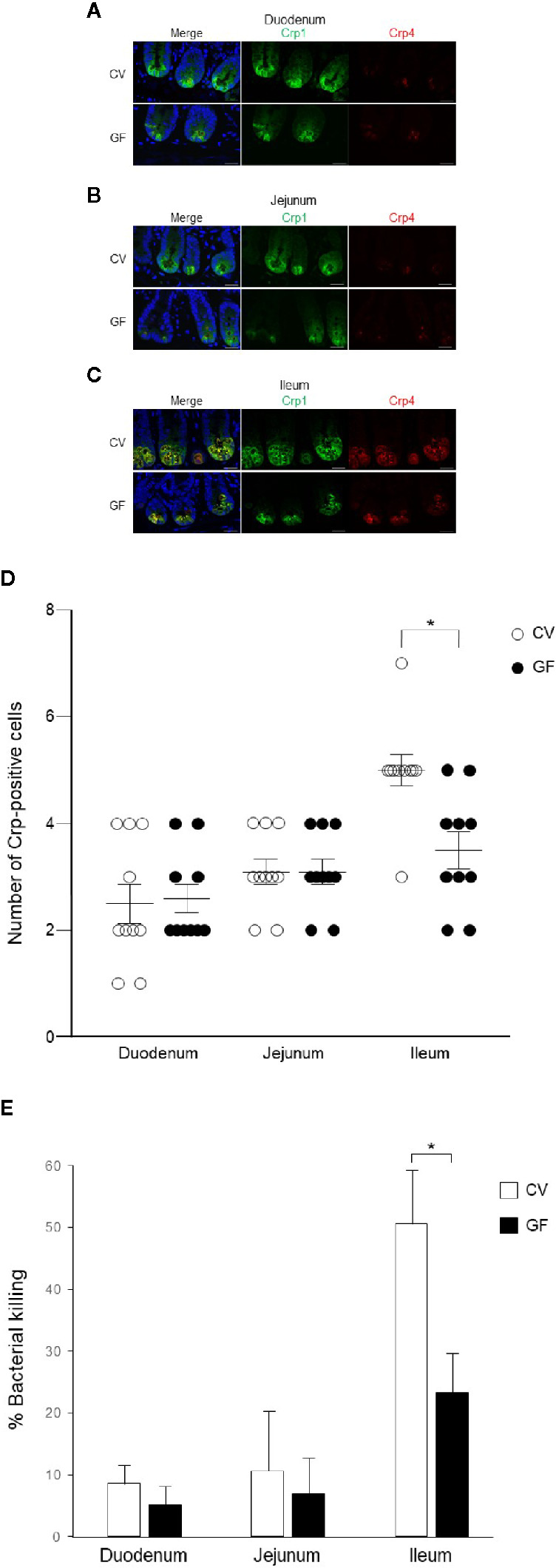
Cryptdin expression and Paneth cell number in the small intestine and bactericidal activities of Paneth cell secretions of conventional and germ-free mice. Representative immunofluorescent staining images for Crp1 (green) and Crp4 (red) in the duodenum **(A)**, the jejunum **(B)**, and the ileum **(C)** of conventional (CV) and germ-free (GF) mice staining together with 4’,6-diamidino-2-phenylindole (DAPI) (blue). Scale bars indicate 20 μm. Number of Crp-positive Paneth cells in single crypt of duodenum, jejunum, and ileum in CV and GF mice **(D)**. n = 10 for each, mean ± SD, *p < 0.05. Percentage of bacterial killing of Paneth cell secretions in *ex vivo* bacterial infection assay in CV and GF mice. n = 3 each, mean ± SD, *p < 0.05 **(E)**.

We further conducted immunohistochemistry of Crp1 and Crp4 in the small intestine of germ-free mice to test whether the intestinal microbiota affect cryptdin expression. We compared cryptdin isoform expression between conventional and germ-free mice, and both Crp1 and Crp4 expressions in each site of the small intestine in germ-free mice were decreased compared to those of conventional mice (**Figures 4A–C**). The number of Crp-positive cells, i.e., Paneth cells in germ-free mice was significantly decreased in ileum compared to that in conventional mice (**Figure 4D**). In contrast, the number of Crp-positive cells in both duodenum and jejunum unchanged in germ-free mice and conventional mice.

Finally, we tested bactericidal activities from each of 1,000 crypt-derived Paneth cell secretion of conventional and germ-free mice from duodenum, jejunum, and ileum stimulated *ex vivo* by *S. typhimurium*. Secretions from duodenum, jejunum, and ileum of conventional mice elicit 8.6, 13.6, and 53.7% killing activities against the bacteria, respectively and those of germ-free mice elicit 3.1, 6.9, and 25.2%, respectively (**Figure 4E**). Bactericidal activities of ileal secretions were significantly greater than those of duodenum or jejunum in both mice. In contrast, control supernatants of duodenum, jejunum, and ileum elicit no bactericidal activities (3.1, −0.1, and 1.5%, respectively in conventional mice and 2.8, 1.8, and −0.5% in germ-free mice). Ileal Paneth cells of germ-free mice released significantly lower bactericidal activities compared to those of conventional mice (**Figure 4E**).

## Discussion

The granules of Paneth cells are rich in α-defensins, and also it has been known to contain other microbicidal or anti-microbial constituents such as lysozyme, secretory phospholipase A_2_, and angiogenins (25–27). Recently, there have been emerging evidences that α-defensins secreted by Paneth cells serve vital roles in innate enteric immunity and regulating intestinal microbiota in humans and mice (8–10). Therefore, this study focused on mouse Paneth cell α-defensin, cryptdin, and determined mRNA expression of Crp isoforms, Crp1-6 at single crypt level from duodenum, jejunum, and ileum of conventional mice by conducting quantitative PCR. Previous studies addressing quantity of Crp isoform gene expression in the mouse small intestine showed that Crp1 is most abundant and Crp4 is relatively less in the protein extracted from intestinal tissues of ICR mice (15). It has been also reported that mRNA expressions for Crp1 and Crp4 are 1.2 and 16 times higher, respectively, when compared ileum *versus* duodenum by conducting quantitative RT-PCR of total RNA extracted from small intestinal tissues of FVB mouse (28). Expression levels of Crp mRNA of C57BL/6 mice through an optimized set of primers has been reported that marked differences of the expression are found from the duodenum to the ileum as well developmental stage (29). Another report showed that Crp mRNA expression in the ileal tissue was 1.5 to 20 times higher than that of jejunum, and the gene expression levels of each isoform are most abundant in Crp1 and fewest in Crp2 in jejunal and ileal tissues of BALB/c mouse (17). Together with previous findings, by the evidence that we showed here using isolated single crypt that each Crp isoform gene expression has no difference between duodenum and jejunum, whereas that is significantly higher in ileum relative to jejunum, suggesting that Crp mRNA expression is controlled topographically in the small intestine. Especially, gene expression levels of Crp4, which is known to elicit most potent microbicidal activities among other Crp isoforms in ileum were 3 and 46 times higher compared to that in jejunum and duodenum, respectively, indicating that Crp4 is the most topographically controlled among Crp isoforms, consistent with previous report (15, 28, 29). Previous study revealed that Crp1 and Crp5 gene expression levels are almost equal in jejunum and ileum (17). We revealed that the gene expressions of each Crp isoform in duodenum, jejunum, and ileum are same in order, as maximum Crp3 > 6 > 1, 4, 5 > minimum Crp2 in single-crypt level. This study further showed that Crp isoform mRNA expression ratios in duodenal, jejunum, and ileum to GAPDH gene expression were from 0.1 for duodenal of Crp2 as minimum to 112 for ileal of Crp3 as maximum.

In this study, all Crp isoform gene expression was significantly elevated in the ileum compare to the jejunum of conventional mice. In addition, as previously reported, although it was faint but Crp4 peptide was present in the jejunum. Crp-1, -2, -3, and -6 are classified as Crp1-like family by sharing highly homologous primary structure. Among these, the homology is the highest in Crp2 and Crp3 (16), which are known to induce Cl- secretion in opening a hole in the eukaryotic cell membrane (30), showing killing activities against *G. lamblia* (18). Crp2 and Crp3 further elicit Cl^-^ secretion in the intestinal epithelial cells (31). In addition, it has been reported that Crp3 induces inflammatory cytokine secretion (2). In addition, it has been reported that conventional mice showed significantly higher gene expression for all isoforms than germ-free mice, suggesting that the intestinal bacteria affect Crp gene expression (32). We revealed that Crp3 mRNA expression is thirty times higher in the jejunum and the ileum compared to Crp2, suggesting important role in the small intestine of Crp3. It has been reported that Crp4 and 5 have the sterilization activities stronger than Crp1-like-family peptides (12). Importantly, the gene expressions of human Paneth cell α-defensins, HD5 and HD6, in the ileum have been reported to be several times greater compared to those in the jejunum (20).

Intestinal microbiota plays a critical role in maintaining intestinal homeostasis. In this study, we compared Crp1 and Crp4 immunostaining and their function in conventional and germ-free mice. The influence of the intestinal microbiota on Crp expression remains controversial. It has been reported that cryptdin mRNA is equally abundant in germ-free and conventional mice (14), contrary, also reported lower mRNA expression for Crp1 and Crp4 in germ-free mice (28). Our results in peptide level that ileal Paneth cells of germ-free mice express fewer Crps and release decreased bactericidal activities compared to conventional mice suggest that the intestinal microbiota may be partially required for normal Paneth cell function. Further detailed studies are needed to understand the effects of the microbiota on Paneth cell function. Dysbiosis, a state of disrupted the composition or the amount of microbiota reside in the intestine, gives rise to a variety of diseases such as life-style diseases, neurological disorders, and cancers (33–36). Disruption of α-defensin secretion has been known to cause dysbiosis and result in certain diseases such as obesity, Crohn’s disease, and graft-*versus*-host-disease (37–41). In addition, NOD2 mutation leads to a decrease in α-defensin production in Paneth cells in patients with Crohn’s disease (42) and amounts of α-defensin peptides decrease in obesity (43). Severe dysbiosis due to lack of Crps in graft-*versus*-host disease model mice reversed by administration of Wnt agonist R-Spondin1 to restore Paneth cells and their Crps, leading to recovery from dysbiosis and resulting in alleviating the disease condition (44).

It has been difficult to observe detail of the intestinal epithelia and hard to count numbers of Paneth cells in the small intestine using the tissue section. Using isolated small intestinal crypts with confocal microscopy, we showed that in the mouse ileum, not only the number of Paneth cells but the proportion of Paneth cells with Crp are significantly higher, and the number of granules with Crp in Paneth cells is also higher. These results suggest that the Paneth cell number and the Crp expression in Paneth cells are spatially regulated in the small intestine. In this study, the ileal Paneth cells which express maximum for both Crp genes in isolated single-crypt level and Crp antibody reactivities in the small intestine elicited most potent bactericidal activities against *Salmonella*, suggesting that gut innate immunity is spatially well-regulated. Obviously, ileum is close to the large intestine, which harbors a huge number of the intestinal microbiota, so that Crp4 having the most potent bactericidal activities may need to be placed predominantly. In Crohn’s disease, an intractable inflammatory bowel disease, it is known that lesion formation is predominant in the terminal ileum. The amount and the quality of α-defensins in Crohn’s disease have been shedding important insights into the pathogenesis and pathophysiology of the disease (42, 45, 46). Bacterial overgrowth is known to occur not only in the colon but in affected lesions in the terminal ileum in patients with severe ulcerative colitis. It has been reported that α-defensins exert a strong innate immune function in the ileum as well as the large intestine (47–49), suggesting that there may be a relationship between disruption of their functions and pathology of inflammatory bowel disease. Furthermore, not only innate immunity but also symbiosis with the microbiota elicited by Paneth cells in the small intestine have been considered to contribute to maintaining host health and prevent certain diseases (50). In humans, it is known that Paneth cells appear ectopically in response to certain sever chronic inflammation including in gastric mucosa with intestinal metaplasia and colonic mucosa with ulcerative colitis (51). It is also possible that cells adapt to various environmental differences of the lumen of the small intestine, i.e., the intestinal environment. Therefore, it is suggested that spatial control may also be exerted on Paneth cell development and environmental adaptation. Mechanisms controlling the localization of Paneth cell α-defensins in conventional and germ-free mice remain to be determined, and further study is necessary to clarify the underlying mechanisms.

## Data Availability Statement

The datasets presented in this study can be found in online repositories. The names of the repository/repositories and accession number(s) can be found in the article/**Supplementary Material**.

## Ethics Statement

The animal study was reviewed and approved by: The committee on Animal Care and Use at Hokkaido University in accordance with Hokkaido University Regulations of Animal Experimentation.

## Author Contributions

KN developed the conceptual framework of the study, designed the experiments, conducted experiments, data analysis, interpretation, and wrote and reviewed the paper. YY and RF designed and conducted experiments, data analysis, and interpretation. SO, RS, TN, and MK conducted data analysis and interpretation. TA developed the conceptual framework of the study, data analysis, and interpretation, and reviewed the paper.

## Funding

This work was supported by grants from the Japan Society for the Promotion of Science Grant-in-Aid for Scientific Research (C) Grant Number 17K11661 (to KN) and (B) 18H02788 (to TA), and the Center of Innovation Program from the Japan Science and Technology Agency Grant Number JPMJCE1301 (to KN and TA).

## Conflict of Interest

The authors declare that the research was conducted in the absence of any commercial or financial relationships that could be construed as a potential conflict of interest.

## Supplementary Material

Supplementary Figure 1 Ratio of cryptdin mRNA expression levels in the isolated single crypt of duodenum, jejunum, and ileum against GAPDH **(A)**. Ratio of mRNA expression of each Crp isoform in duodenum and jejunum against ileum **(B)**.

Supplementary Table 1 Primer pairs for cryptdin isoforms and GAPDH.
