# Supplementary figures and images for: Expression and Localization of Paneth Cells and Their α-Defensins in the Small Intestine of Adult Mouse
Source: Front Immunol. 2020 Oct 13;11:570296. doi: 10.3389/fimmu.2020.570296 (PMC7590646; doi:10.3389/fimmu.2020.570296)

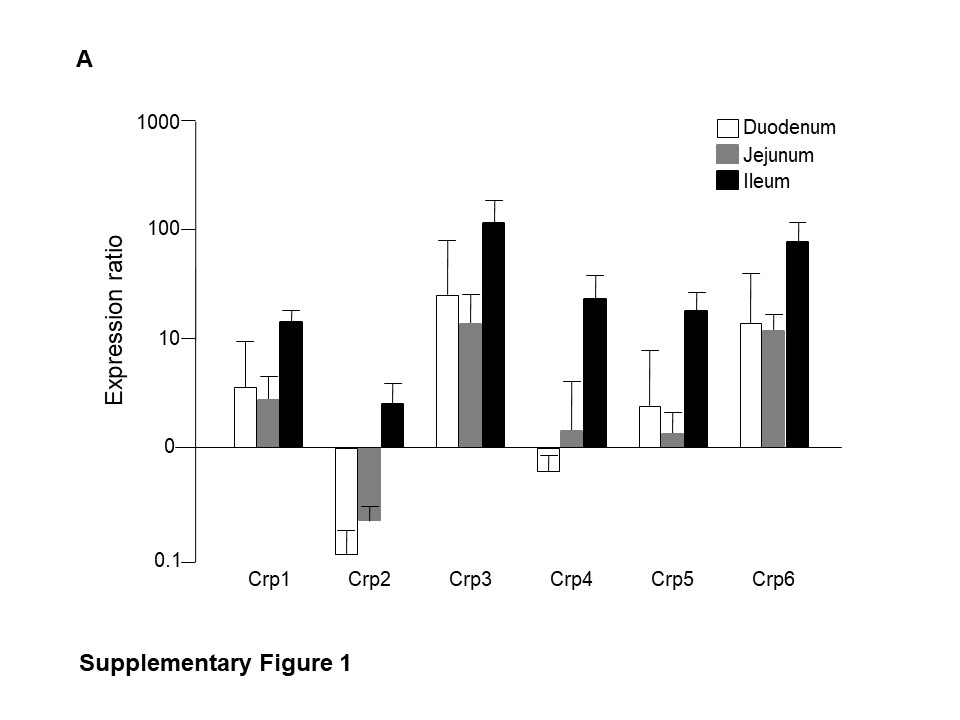

Supplement: Supplementary Figure 1 — Ratio of cryptdin mRNA expression levels in the isolated single crypt of duodenum, jejunum, and ileum against GAPDH (A). Ratio of mRNA expression of each Crp isoform in duodenum and jejunum against ileum (B). [file DataSheet_1.zip › Supplementary Figure 1A.JPEG]

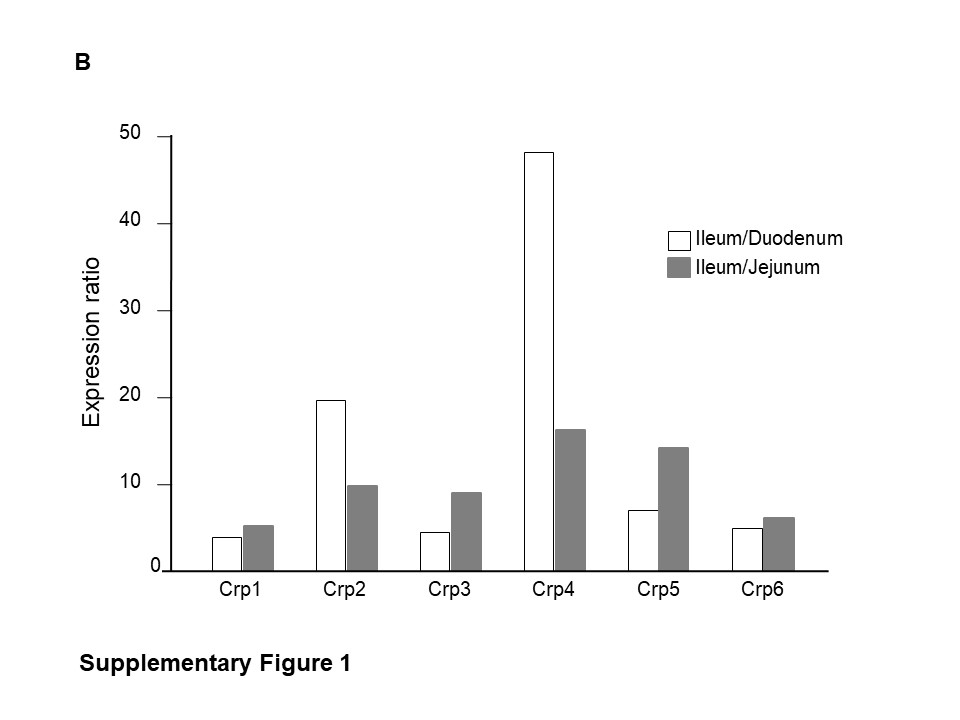

Supplement: Supplementary Figure 1 — Ratio of cryptdin mRNA expression levels in the isolated single crypt of duodenum, jejunum, and ileum against GAPDH (A). Ratio of mRNA expression of each Crp isoform in duodenum and jejunum against ileum (B). [file DataSheet_1.zip › Supplementary Figure 1B.JPEG]

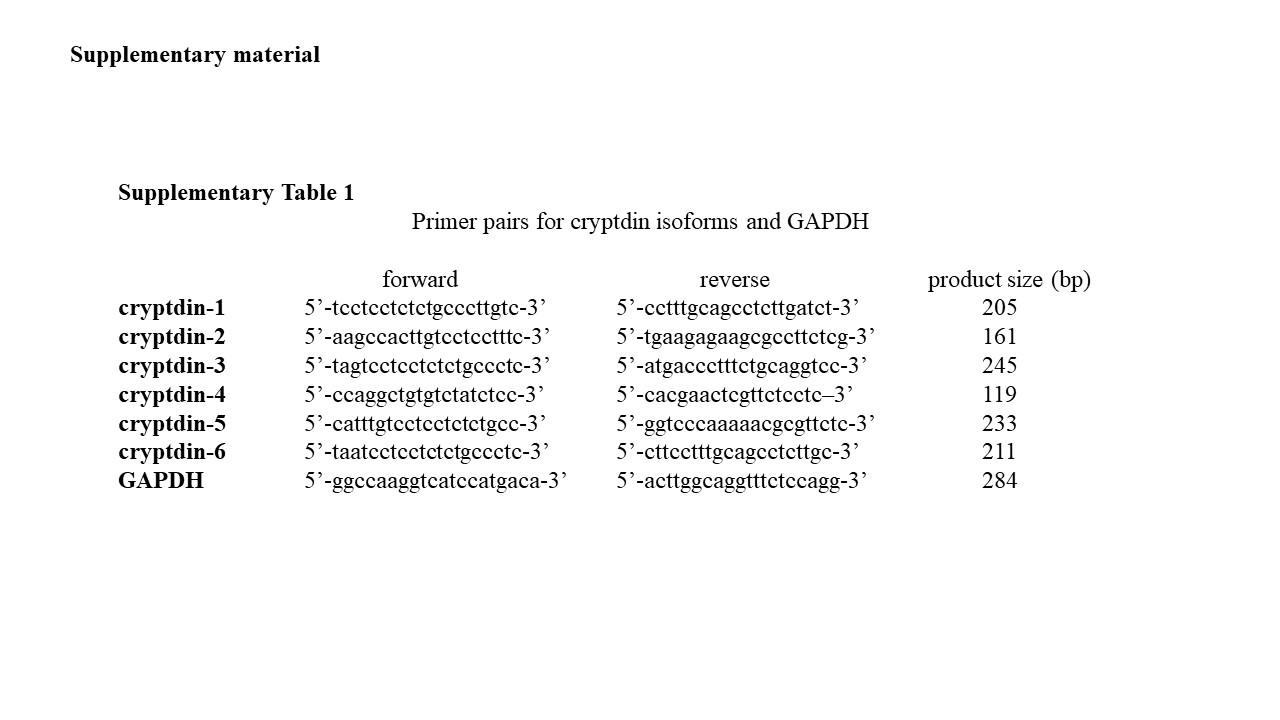

Supplement: Supplementary Figure 1 — Ratio of cryptdin mRNA expression levels in the isolated single crypt of duodenum, jejunum, and ileum against GAPDH (A). Ratio of mRNA expression of each Crp isoform in duodenum and jejunum against ileum (B). [file DataSheet_1.zip › Supplementary Table 1.JPEG]
